# Strategic Isolation of a Polyoxocation Mimicking Vanadium(V) Oxide Layered-Structure by Stacking of [H_2_V_2_O_8_]^4−^ Anions Bridged by (1,4,7-Triazacyclononane)Co(III) Complexes

**DOI:** 10.3389/fchem.2018.00375

**Published:** 2018-08-28

**Authors:** Sugiarto, Keisuke Kawamoto, Yoshihito Hayashi

**Affiliations:** Department of Chemistry, Kanazawa University, Kanazawa, Japan

**Keywords:** cationic oxide cluster, protecting group, hydrogen bonding, 1, 4, 7-triazacyclononane (= tacn), divanadate anion, Cobalt(III)

## Abstract

An isolation of a vanadium(V) oxide cluster mimicking V_2_O_5_ layered structure was achieved formulated as [{Co(tacn)}_4_V_4_O_12_(OH)_4_]^4+^ (**1**) (tacn = 1,4,7-triazacyclononane). From the ^51^V NMR spectra of the reaction mixtures, we optimized the reaction condition in terms of a molar ratio of VO_4_^3−^ and [Co(tacn)(H_2_O)_3_]^3+^ as well as a pH value. Cluster **1** is stable in a wide range of pH values from 1.5 to 8.0, and the presence of multiple hydrogen bondings in the structure is a unique feature. In the X-ray analysis of cluster **1**, the V⋯ V distances are classified into two groups of relatively shorter distances (2.978(1) Å) and longer interactions (3.554(1) Å), and it is a good model of the substructure of V_2_O_5_ bulk material. As far as we know, this is a first example of an isolation of mixed-metal cluster including a unit of V_2_O_5_ structure by a stack of two layers of [H_2_V_2_O_8_]^4−^, although cubic V_4_O_4_ cubane-type clusters are well known. The solid sample of compound **1-Cl** and **1-Br** shows reversible thermochromic behavior accompanied by crystal to amorphous transformation upon hydration-dehydration process.

## Introduction

Metal oxide clusters have attracted much attention because of their relevance to water splitting catalysts with their interesting properties such as redox, magnetism, and reactivity (Pope, 1983; Sessoli et al., 1993; Saha et al., 2011; Ye et al., 2018). To study structure-property relationships, a precise structural control of the oxide cluster is necessary, for example, to survey the interaction between substrates and active sites on metal oxide surfaces (Isobe and Yagasaki, 1993). As one of the important metal oxides, vanadium(V) oxides, V_2_O_5_, has been attracted much attention in terms of oxidation catalysts and biological activities on the oxide surfaces (Al-Qatati et al., 2013). Various vanadium(V) oxide clusters such as polyoxovanadates as mimics of their oxide surfaces also have significance for a construction of better practical materials, because the screening of the model complexes based on the proposed mechanism are possible. To realize their oxide surface structures as model clusters, a synthetic chemistry of anionic oxide clusters of polyoxomolybdate, polyoxotungstate, or polyoxovanadate have been developed in the past decade. Some of the attempts to control oxide cluster structures have been achieved using organometallic capping ligand to prevent the formation of infinitely condensed oxide precipitates and the capping ligands restrict an unlimited condensation and facilitate a formation of an oligometric oxide core (Hayashi et al., 1988; Do et al., 1991; Proust et al., 1993; Takara et al., 1997; Artero et al., 2000, 2001; Villanneau et al., 2003; Boujday et al., 2007).

Another approach to control the structure of oxide clusters is utilizing hydrogen bondings for the stabilization of a cluster unit. Hydrogen bonding interactions in inorganic metal complexes have been receiving attention because they have a potential to induce very interesting properties such as spin flipping (Matheu et al., 2015), proton coupled electron transfer (PCET) (Yikilmaz et al., 2002), low overpotential for essential redox catalysts (Matheu et al., 2017), and oxygen activation in bioinorganic chemistry (Shook and Borovik, 2008). Recently, the importance of intermolecular hydrogen bondings to enable multi-electron-multi-proton transfer in artificial Co_4_O_4_ cluster system was demonstrated for a water-splitting reaction (Olshansky et al., 2018).

**Scheme 1:**
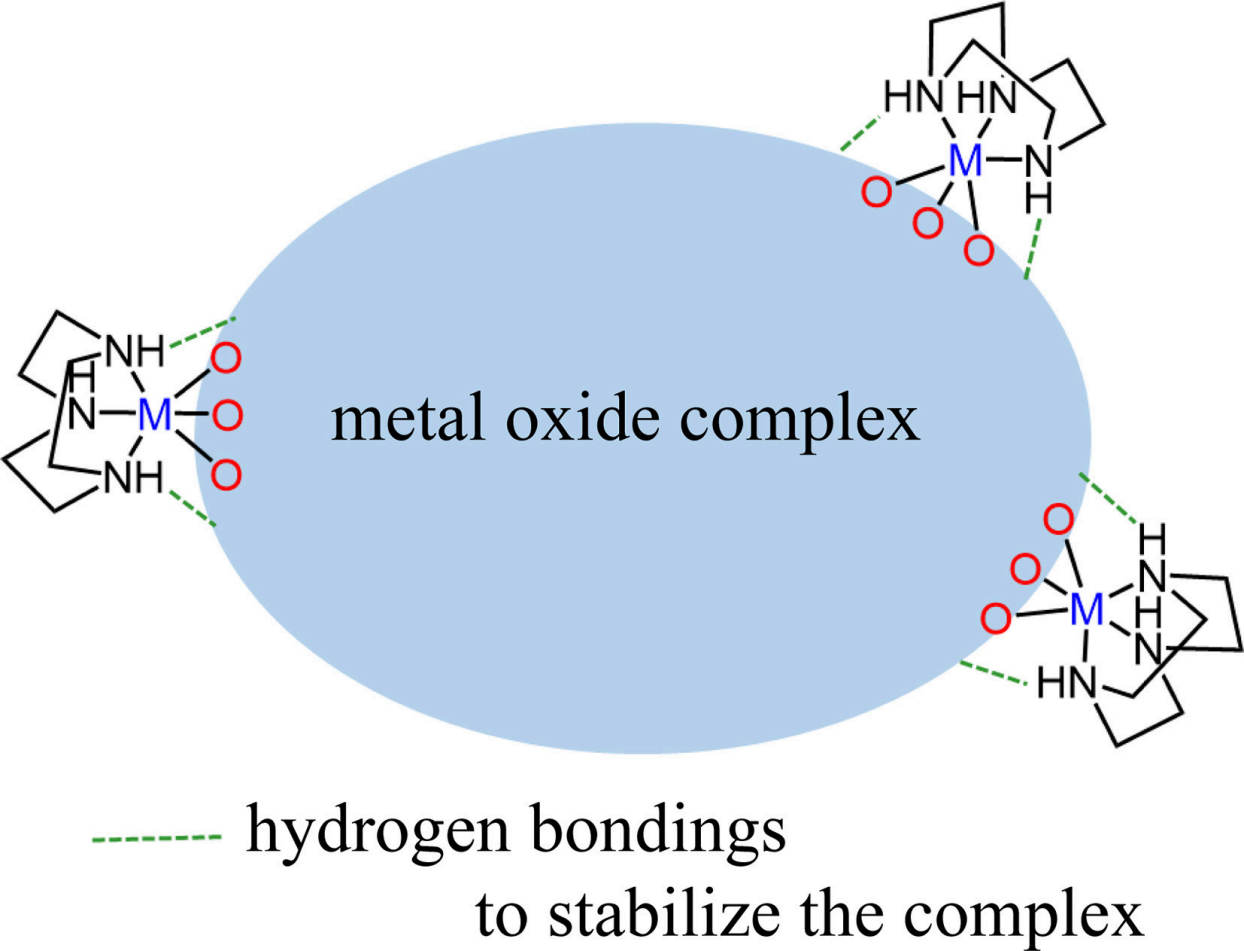
Schematic scheme to stabilize metal oxide complexes protected by tacn complexes with a hydrogen bonding wrapping.

Our focus is a development of a new synthetic method for metal oxide complexes by using inert coordination complexes as a protecting group for the termination of a polyoxoanion growth as well as gaining solubility in water (Scheme 1). Our strategy is using coordination chemistry of *fac*-{Co(tacn)}^3+^ (tacn = 1,4,7-triazacyclononane) (Chaudhuri and Wieghardt, 2011) to induce a following two-fold synergistic effect: (1) the inert *fac*-{Co(tacn)}^3+^ units have a role to terminate the cluster core by capping the ends of oxide units, and (2) NH groups on tacn ligands offer intramolecular hydrogen bondings to stabilize the cluster structures while attaining solubility in water.

**Figure 1:**
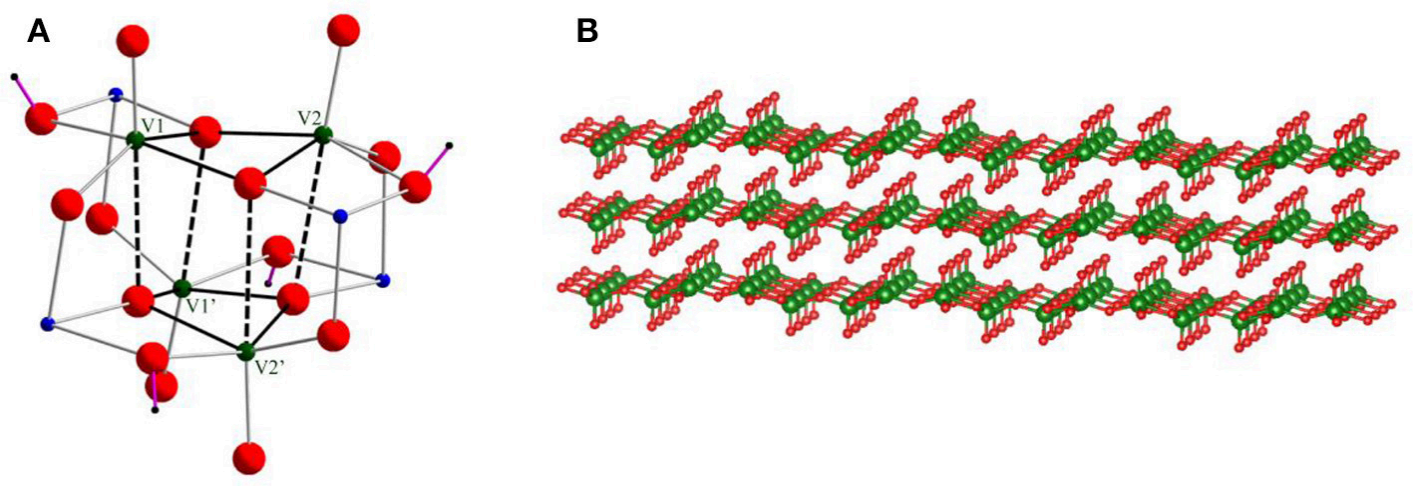
**(A)** Skeletal structure of cationic cluster **1**. Green, blue, and red balls show vanadium, cobalt, and oxygen atoms, respectively. Purple lines are OH bonds. The tacn ligands on Co(III) centers are omitted for clarity. **(B)** V_2_O_5_ layered structure.

Based on the strategy as shown in Scheme 1, we have studied successful stabilization of oxide clusters of molybdenum(VI), vanadium(V), and other various transition metal ions by using *fac*-{Co(tacn)}^3+^ capping units. Regarding to the molybdenum(VI) cluster chemistry protected by the capping unit, we have reported a neutral species of [{Co(tacn)}_2_Mo_3_O_12_] and two cationic species of [{Co(tacn)}_4_H_2_Mo_7_O_27_]^2+^ and [{Co(tacn)}_4_H_3_Mo_4_O_17_]^5+^ (Sugiarto et al., 2018). All these clusters have a common structural building block of [{Co(tacn)}_2_Mo_3_O_13_], and the structural similarity allows us to figure out the method to achieve the controlled structural transformations among them by adjusting the pH conditions for the syntheses. In this paper, we report a synthesis of a distorted cubane-like V_4_O_4_ oxide cluster capped by four *fac*-{Co(tacn)}^3+^ units, in the formula of [{Co(tacn)}_4_V_4_O_12_(OH)_4_]^4+^ (**1**) (Figure 1). By the reaction of *fac*-[Co(tacn)(H_2_O)_3_]^3+^ and vanadate anion in water, we demonstrated a rare example of the synthesis of cationic oxide cluster from vanadate anionic core (Šimuneková et al., 2013), although some neutral clusters bearing vanadium(V) oxide moiety have been previously reported using 4,4′-^*t*^Bu-bpy as organic neutral capping groups (Kodama et al., 2016). Cluster **1** possesses intramolecular hydrogen bondings to stabilize the cluster core based on NH groups from tacn ligands. Multinuclear NMR measurements reveal cluster **1** is stable in a wide range of pH values. In addition, cluster **1** exhibits reversible thermochromic behavior accompanied by crystal to amorphous transformation upon hydration-dehydration process.

## Experimental

### Materials and methods

Tacn and [Co(tacn)(H_2_O)_3_](CF_3_SO_3_)_3_·H_2_O were prepared according to the reported procedures (Wieghardt et al., 1979; Galsbøl et al., 1996). The other chemicals were purchased and used as received. NMR spectra were recorded using a JEOL Spectrometer (400 MHz). FT-IR spectra were recorded using Jasco FT/IR-4100 in KBr pellets. UV–Vis solution absorption and solid state reflectance spectra were recorded using a Hitachi U-3500 spectrometer. CHN elemental analysis was performed by the Research Institute for Instrumental Analysis at Kanazawa University. X-ray powder diffraction (XRD) pattern was recorded using D8 ADVANCE eco.

### Syntheses

[Co_4_V_4_O_12_(OH)_4_(tacn)_4_](CF_3_SO_3_)_4_ (**1-OTf**). Na_3_VO_4_ (24.0 mg, 0.13 mmol) and [Co(tacn)(H_2_O)_3_](CF_3_SO_3_)_3_·H_2_O (80.0 mg, 0.11 mmol) were mixed in water (2.0 mL). The mixture was acidified by 0.22 M of triflic acid adjusted to pH 7.0. The crystals formed were harvested after 2 days. Yield: 30.5 mg (65% based on Co). Anal. Calcd., for [Co_4_V_4_O_12_(OH)_4_(C_6_H_15_N_3_)_4_](CF_3_SO_3_)_4_·4H_2_O: C, 17.84; H, 3.86; N, 8.92. Found: C, 17,76; H, 3.68; N, 8.95%. ^1^H NMR (D_2_O, δ): 4.06 (m, 4H), 3.48 (m, 4H), 3.26 (m, 4H), 3.00-2.65 (m, 24H), 2.52 (m, 4H), 2.40 (m, 4H), 2.26 (m, 4H) ppm. ^51^V NMR (D_2_O, δ): −380 ppm. ^59^Co NMR (D_2_O, δ): 9550 ppm. UV–Vis λ_MAX_ / nm (ε / M^−1^ cm^−1^) (H_2_O): 529.5 (754), 401.0 (4086).

The bromide (**1-Br**), chloride (**1-Cl**), and perchlorate (**1-ClO**_4_) salts were also obtained by adding four equivalents of potassium bromide, sodium chloride, and sodium perchlorate to the reaction mixture, respectively. From IR and NMR spectra of the products, the cluster integrity in the solution was confirmed (Figure S1).

### X-ray crystallography

X-ray analyses of **1-ClO**_4_, **1-Br**, and **1-Cl** were performed. The crystallographic parameters are shown in Table 1. All measurements were performed at 90K by using a Bruker D8 VENTURE diffractometer with graphite monochromated Cu-Kα radiation (λ = 1.54178 Å). The data reduction and absorption correction were performed on *APEX3* program (Bruker, 2016). The structural analyses were performed on *APEX3* and *shelxle* software (Hübschle et al., 2011). The structures were solved by SHELXT (Sheldrick, 2015b), and the refinements were performed using SHELXL-2014 program (Sheldrick, 2015a). In the refinement of **1-ClO**_4_, the twin law (-1 0 0 0−1 0 1 0 1) was used to reduce R_1_ value from 11.15 to 9.51. CCDC reference numbers 1835286–1835288.

**Table 1:** Crystallographic data for **1-Cl**, **1-Br**, and **1-ClO**_4_.

	**1-Cl**	**1-Br**	**1-ClO_4_**
Formula	C_12_H_32_Cl_2_Co_2_N_6_O_13_V_2_	C_12_H_32_Br_2_Co_2_N_6_O_13_V_2_	C_*l*2_H_30_Cl_2_Co_2_N_6_O_18_V_2_
fw	759.07	848.00	837.06
Crystal system	Tetragonal	Tetragonal	Monoclinic
Space group	*P*$\overset{\bar{\;}}{4}$2_1_*c*	*P*$\overset{\bar{\;}}{4}$2_1_*c*	*P*2_1_/*n* (#14)
*a*, Å	14.5508(4)	14.7055(3)	15.2827 (8)
*b*, Å	14.5508(4)	14.7055(3)	14.9893 (8)
*c*, Å	12.4185(4)	12.4952(3)	25.1980 (13)
α, deg	90	90	90
β, deg	90	90	106.744 (3)
γ, deg	90	90	90
*V*, Å^3^	2629.32 (17)	2702.11 (13)	5527.5 (5)
*Z*	4	4	8
*R*; *wR_2_* (all data)	0.0278; 0.0725	0.0278; 0.0671	0.1074; 0.2904
*R_1_*	0.0258	0.0260	0.0951

## Results and discussion

### Synthesis

**Figure 2:**
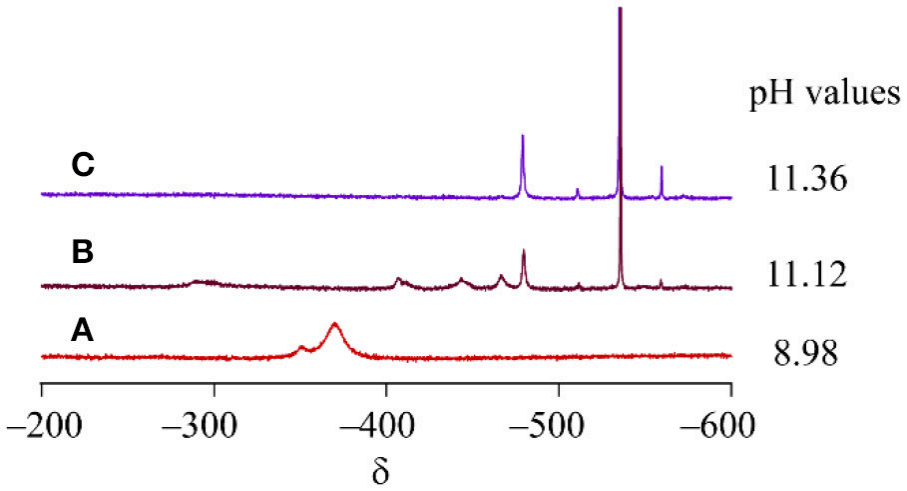
^51^V NMR spectra of the reaction mixture of [Co(tacn)(H_2_O)_3_]^3+^ and VO_4_^3−^. The molar ratio is **(A)** 1:1, **(B)** 0.5:1, **(C)** 0.25:1, respectively.

Cluster **1** was synthesized by the reaction of VO_4_^3−^ and [Co(tacn)(H_2_O)_3_]^3+^ in water with a 1:1 molar ratio. To monitor the formation of cluster **1**, ^51^V NMR spectra of the reaction mixtures. were measured at different molar ratios of the starting materials. When 0.25 or 0.50 equivalence of [Co(tacn)(H_2_O)_3_]^3+^ to VO_4_^3−^ was added to the reaction mixture, a signal for cluster **1** was not found and only a strong peak from VO_4_^3−^ at −535 ppm was observed, but there are unidentified peaks that was presumed to be an intermediate species (Figure 2). After adding 1.00 equivalence of [Co(tacn)(H_2_O)_3_]^3+^ to VO_4_^3−^, the strong peak from cluster **1** at −370 ppm was observed and the peak from the starting material was disappeared (Figure 2). Reaction of [Co(tacn)(H_2_O)_3_]^3+^ and VO_4_^3−^ with a 1.5:1 molar ratio leads to a rapid crystallization of cluster **1**. This observation suggests that formation of cluster **1** is kinetically favored. The variation of the molar ratio of the starting material also affected the pH value of the solution. Formation of cationic cluster **1** can be expressed as:

$$\begin{array}{l} 4{\left[\text{Co}\left(\text{tacn}\right){\left({\text{H}}_{\text{2}}\text{O}\right)}_{\text{3}}\right]}^{3+}(aq)+{\text{4VO}}_{4}{}^{3-}(aq)+{\text{4H}}^{+}(aq)\\ \to \;{\left[{\text{Co}}_{4}{\text{V}}_{4}{\text{O}}_{12}{\left(\text{OH}\right)}_{4}{\left(\text{tacn}\right)}_{4}\right]}^{4+}(aq)+{\text{12H}}_{2}\text{O}(l) \end{array}$$

From the above equation, the addition of acids is necessary. In fact, we optimized the best synthetic condition for the synthesis of cluster **1** with 1:1 molar ratio at pH 7 by addition of trifluoromethanesulfonic acid.

### Structure description

The chloride salt of **1** crystallizes in a tetragonal crystal system. The crystallographic parameters are shown in Table 1. The structural feature of **1-Cl** is similar to that of **1-Br** and **1-ClO**_4_. Therefore, we describe the structural features of V_4_O_4_ unit based on the structure of **1-Cl** in this section.

Figure 1A shows the skeletal structure of cationic cluster **1**. Cluster **1** is composed of a V_4_O_12_(OH)_4_^8−^ core capped by four {Co(tacn)}^3+^ complexes. Bond valence sum calculations indicated that the four cobalt and vanadium centers are assigned to III and V oxidation states, respectively. It also reveals protonations on four doubly bridged oxygen ligands between Co(III) and V(V) centers, i.e. the ligands are hydroxyl groups. The hydroxyl bridging ligands play an important role to construct intramolecular hydrogen bonding interactions. Four Co(III) centers are six-coordination-mode, and the bond distance of Co(III) and N donors of tacn ligands is in a range from 1.932(3) Å to 1.953(3) Å and it is consistent to the previous reported values (Berseth et al., 2000). For the geometry of V(V) centers, if the weak interactions at the trans-position of V = O [2.564(3) Å] are ignored, four V(V) centers can be regarded as a five-coordination-mode with one terminal oxygen ligand (V = O) and four bridged ligands. Thus, the geometry around V(V) centers can be regarded as a distorted square pyramid and it is also indicated by its geometry index τ of 0.06 {τ is defined as (α-β)/60°, where α = the largest angle and β = the second largest angle. (τ = 0.0 for an ideal square pyramidal. τ = 1.0 for an ideal trigonal bipyramidal.)} (Addison et al., 1984).

Although the tetranuclear V_4_O_12_(OH)_4_^8−^ unit has a cubane-like arrangement, the long distances between [H_2_V_2_O_8_]^4−^ units suggest that the structure is better described as a stack of two [H_2_V_2_O_8_]^4−^ dimers. The V⋯ V distances are classified into two groups of relatively shorter distances (2.978(1) Å) and longer interactions (3.554(1) Å). From the layered structure, we postulated that the structure of cluster **1** can be regarded as a model species which represents a unit of the layered structure of V_2_O_5_ (Figure 1B). Although a cubane type structure of V_4_O_4_ has been previously reported in literature, our structure is quite different from the previous reported cases. In one of the precedented cases, the cubane type cluster was capped by phosphate bridging ligands (Shi et al., 2004). Although the phosphate ligand bridges the V centers in tetrahedral coordination mode, the Co(III) centers on Co(tacn) moieties have octahedral one. As a result, the cluster core geometry protected by the phosphate ligand is totally different to that of **1-Cl**. Dey et al. also described the phosphate ligand coordinated V_4_O_4_ structure linked by two copper(II) 1,10-phenanthroline complexes (Dey et al., 2012). This V_4_O_4_ structure is similar to that of **1-Cl**, but the cubane structure is more tightly packed and the intra-layer distance is much more shorter ca. 3.4 Å (Table 2). Our [H_2_V_2_O_8_]^4−^ layered structure is representing the V_2_O_5_ stacked structure by the influence of a bridging metalloligand of {Co(tacn)}^3+^ which enable to keep the layered structure in the cluster by forming hydrogen bondings from NH groups.

**Table 2:** Distances (Å) between vanadium centers. The serial numbers of vanadium centers are shown in Figure 1.

	**V1–V2**	**V1 ⋯ V1' (V2 ⋯ V2')**
1-Cl	2.978 (1)	3.554 (1)
1-Br	2.992 (2)	3.563 (2)
1-ClO_4_	2.998 (2) 2.994(2)	3.442 (2) 3.431 (2) 3.427 (2) 3.425 (2)
Dey et al., 2012	2.7618 (9)	3.311 (1)
Shi et al., 2004	2.762 (1) 2.816 (1)	3.302 (1) 3.332 (1) 3.354 (2) 3.362 (2)

**Figure 3:**
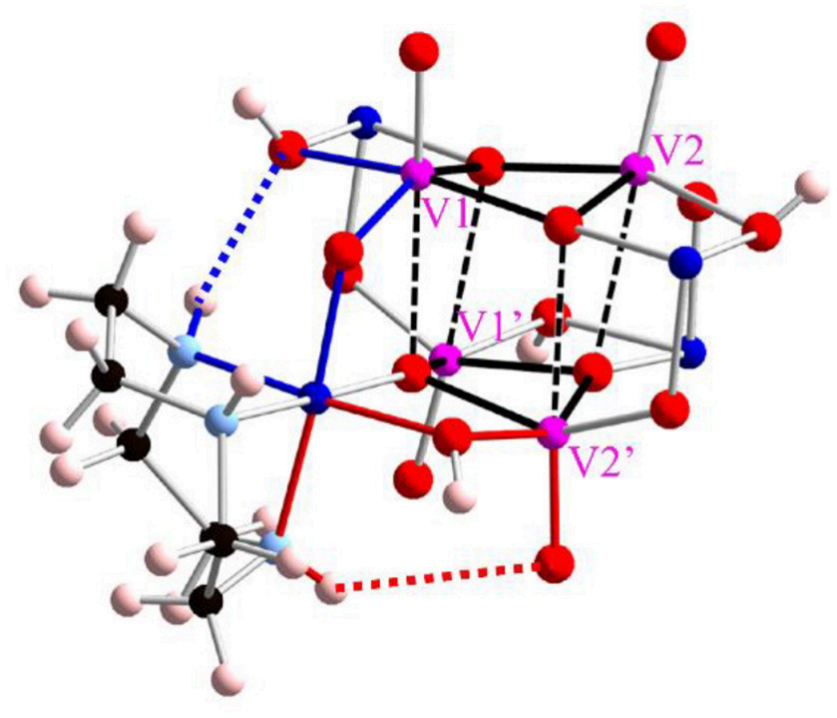
Intramolecular hydrogen bonding interactions with tacn ligands. Pink, blue, and red balls show vanadium, cobalt, and oxygen atoms, respectively. The red and blue dotted lines show N–H·O (terminal) and N–H·OH (μ_2_), respectively. For the simplification, only one tacn ligand is shown, and three other tacn ligands are omitted.

There are two types intramolecular hydrogen bonding interactions. One is between NH group and terminal oxygen ligand with a donor-acceptor distance of 3.431(5) Å, another is an interaction between NH group and bridged hydroxo ligand as a donor-acceptor distance of 2.878(5) Å (Figure 3). All hydrogen bonding interactions are orienting to form the structure of a windmill-shaped geometry with S_4_ symmetry. In crystal packing, counter chloride anions are stabilized by hydrogen bondings with NH groups as well as three crystallization water molecular chains. Regarding to their crystallization water molecules, one water molecule directly interacts with the hydroxido ligands bridged between vanadium(V) and cobalt(III). The packing water molecule allows the linkage between the cluster units by hydrogen bondings at hydroxido group on the cluster. In addition, the water molecule interacted to the hydroxido bridging ligand also binds counter chloride anion via hydrogen bonding. Finally, another water molecule bridges between the counter chloride anion and terminal oxygen ligand on vanadium(V). This crystal packing implies that the alternation of the hydrogen bonding patterns with the crystallization water molecules may relate to the thermochromic behavior. In fact, the desorption of water molecules from the crystal leads to manifest an interesting thermochlomism behavior (vide infra).

### NMR spectroscopy

**Figure 4:**
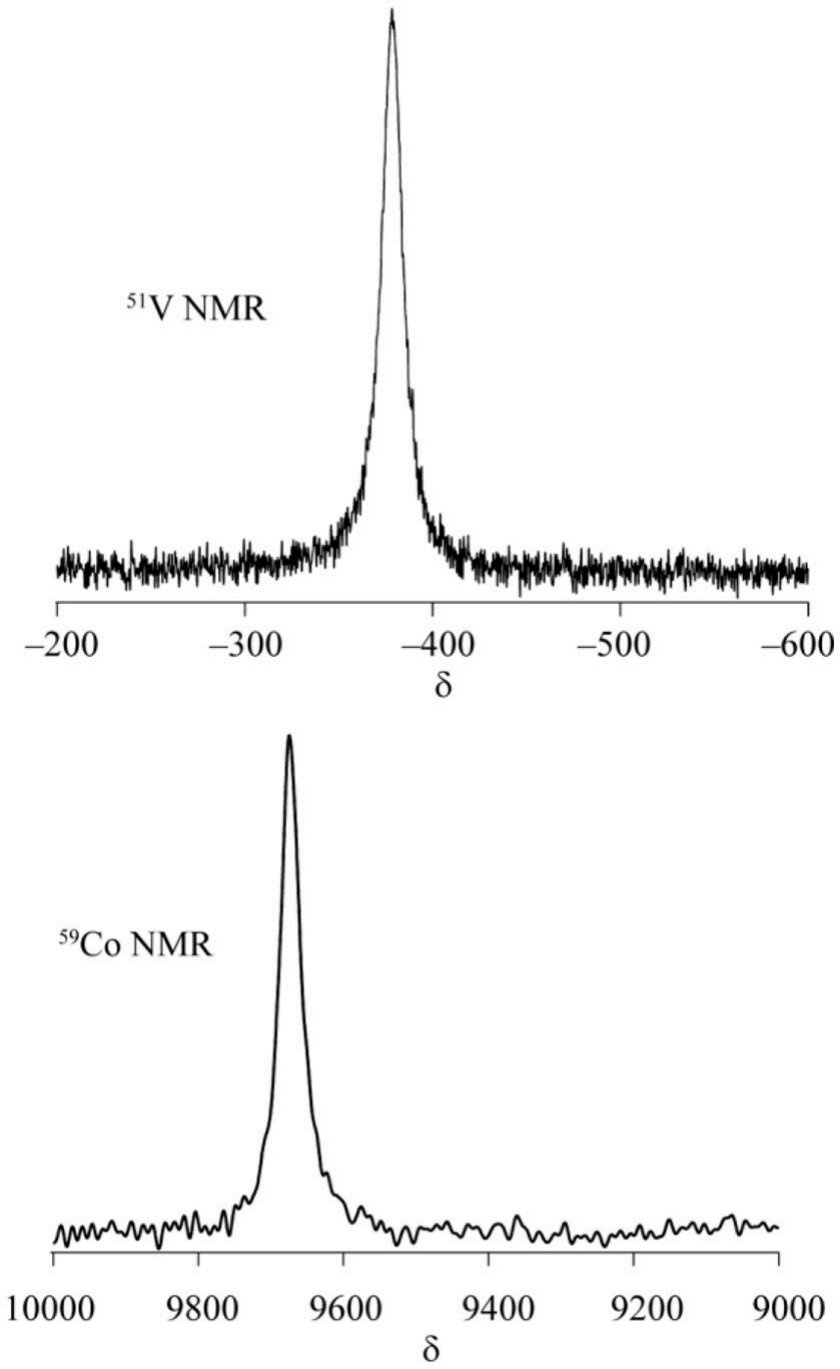
^51^V and ^59^Co NMR spectra of cluster **1** in water at pH = 6.5.

Cluster **1** has four Co(III) and four V(V) centers which are diamagnetic transition metal ions. Therefore, the synthetic procedure of cluster **1** was optimized by monitoring NMR spectra (described in above), and the stability of cluster **1** was also evaluated using NMR technique. Figure 4 shows ^51^V and ^59^Co NMR spectra of cluster **1** at pH = 6.5. In the measurements of ^51^V and ^59^Co nuclei, only one signal in each spectrum was observed at −380 ppm and 9,650 ppm, respectively, implying that the structure of cluster **1** as described in the above section is maintained even in solution, because the chemical environments of each metal atom is equivalent. In addition, the NMR signals of cluster **1** suggest the structural integrity is maintained in the pH range from pH = 1.5 to pH = 9 (Figure S2).

The tacn ligand is a good indicator to monitor the symmetry of the cluster in solution because ^1^H NMR signals on tacn give stereo-specific signals according to the symmetry of the structure. Concerning the methylene proton signals in ^1^H NMR spectrum in D_2_O, six well-separated signals were observed at 4.06, 3.48, 3.26, 2.52, 2.40, and 2.26 ppm, whereas the rest of signals corresponding to six protons are overlapped between 3.00 and 2.65 ppm. The signal intensities are 1:1:1:1:1:1:6. Thus, all twelve protons of a tacn moiety were observed independently, indicating that the capping of {Co(tacn)}^3+^ to V_4_O_12_(OH)_4_^8−^ core are maintained even in solution.

The important feature to bind the layered structure is the formation of hydrogen bondings. Two NH protons on tacn ligand are loosely linked to terminal oxygen ligand or crystallization water molecule by hydrogen bonding, whereas another NH proton is strongly interacted with hydroxo bridging ligand. As a consequence, the observation of hydrogen-deuterium (H/D) exchange reactions allows us to investigate difference of the strength of the hydrogen bondings. The ^1^H NMR signals from OH and NH protons show an important information due to its H/D exchange reactions. To obtain H/D exchange reaction profile, ^1^H NMR spectrum of cluster **1** was measured in DMSO-*d*_6_ as an aprotic solvent (Figure S3). There are four kinds of hydrogen bondings: three types of NH protons (7.29, 6.15, 5, 69 ppm) arose from independent tacn environment and the fourth type of proton from OH protons (3.69 ppm). After adding D_2_O to the DMSO-*d*_6_ solution of cluster **1**, the intensities of the signals for NH and OH at 7.29 and 6.15 ppm are rapidly decreased while the signal at 5.69 ppm is slowly decreased (Figure S4). It is noteworthy that the observation suggests the inner core hydrogen bondings are slower to exchange and they are utilized to firmly support the cluster frameworks.

### Chromism behavior

**Figure 5:**
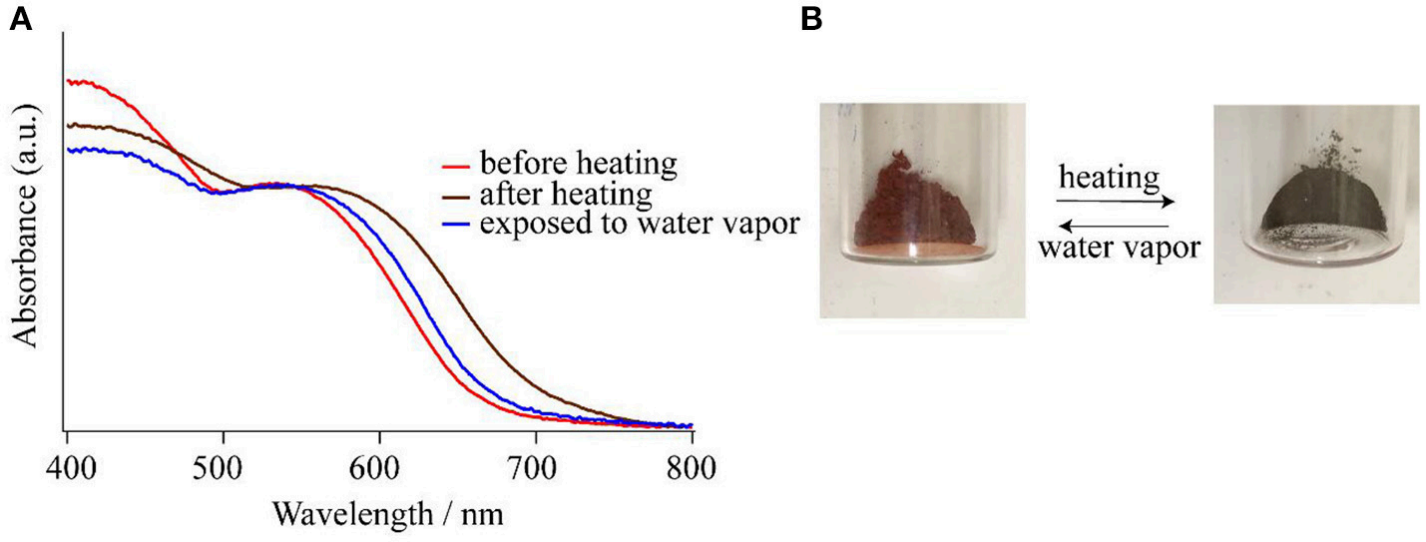
**(A)** Reflectance spectra of **1-Br** before heating (red line), after heating at 150°C (brown line), and after exposing to water vapor (blue line). **(B)** Photographs of the photochromism of **1-Br**.

**Figure 6:**
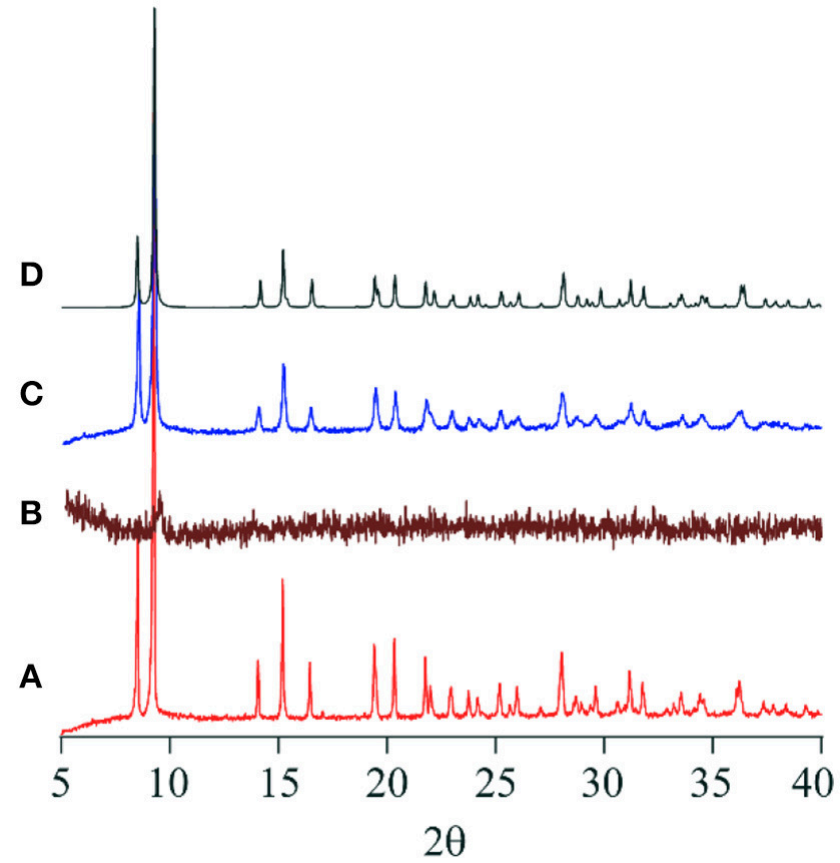
XRD powder pattern of **(A) 1-Br** before heating, **(B) 1-Br** after heating at 150°C, **(C) 1-Br** after exposing to water vapor, and **(D)** simulated from the SC-XRD result of **1-Br**.

As shown in the section of *Structure description*, the counter anions of Cl^−^ and Br^−^ in **1-Cl** and **1-Br** are interacted through hydrogen bonding networks with NH groups and crystallization water molecules, respectively. The crystal packings of **1-Cl** and **1-Br** are identical, because they are isomorphous (Table 1). Therefore, it is expected that these species should show similar behaviors in crystalline phases. In fact, compounds **1-Cl** and **1-Br** show similar thermochromic behaviors by heating at 150°C. In this section, the thermochromic behavior of **1-Br** is described. Compound **1-Br** shows red color, and the solid state reflectance spectrum exhibits an absorption around 550 nm assigned to Co(III) d-d transition, and the feature is identical to solution state absorption spectrum. After heating **1-Br**, the color shift was observed from red to greenish brown with bathochromic shift of the reflectance spectra (Figure 5). Exposure of the greenish brown powder to water vapor retrieves the red **1-Br** salt. It is noteworthy that the color changings by heating and exposing water vapor occur repeatedly with phase transitions between crystal and amorphous state. Powder X-ray diffraction (PXRD) pattern of **1-Br** is identical to the simulated result from the single-crystal X-ray analysis (Figure 6). After changing the color from red to greenish brown by heating, the crystallinity of the sample was lost, resulting the loss of Bragg peaks in PXRD (Figure 6). The peaks of **1-Br** were appeared again when the amorphous sample was exposed to water vapor (Figure 6). These reversible thermochromism is an interesting feature of these clusters and we suggest the presence of hydrogen bondings in the cluster unit is reflected to the reversible change on the alternation of the coordination environment of Co(III) centers.

Interestingly, no thermochromic behavior was observed in the case of **1-OTf**. Although a single crystal X-ray analysis of **1-OTf** was not successful, the crystal system is expected to be different from **1-Cl** and **1-Br** because of the difference of the anion shape. Thus, the absence of the behavior is due to the different hydrogen bonding interactions among the cluster, the counter anions, and the crystallization water molecules in **1-OTf**. We did not examine the property in the case of **1-ClO**_4_, due to the risk of the explosion for perchlorate sample without crystallization water molecules.

## Conclusions

A V_4_O_4_ oxide cluster in the formula of [{Co(tacn)}_4_V_4_O_12_(OH)_4_]^4+^ (**1**) was synthesized utilizing the following synergetic effects: (1) termination of vanadium(V) oxide cluster by Co(tacn) protecting groups, and (2) hydrogen bonding interactions from NH groups on tacn ligands. The V_4_O_4_ core can be regarded as a stacking of [H_2_V_2_O_8_]^4−^ cluster units, mimicking a substructure of V_2_O_5_ oxide. The stacking geometry of cluster **1** is maintained by Co(tacn) termination as well as hydrogen bondings within the cluster units.

Our strategy of a termination of polyoxo-anions by coordination complexes enhances the possibility to isolate a substructure of metal oxido species and take a snapshot of interesting intermediate species in aqueous solution. This study opens the new way to isolate versatile mixed-metal clusters that have an intermediate structure with water solubility by the utilization of hydrogen bonding stabilization and termination by the capping groups. The synthetic strategy defines here is applicable to a wide range of mixed-metal clusters because there are unlimited combination of potential capping groups and oxide cluster cores. Unique feature of this study is that by adding the cationic protecting group, anionic polyoxometalates come to gain exceeding positive charges from the cationic complexes, and the change from polyoxoanions to polyoxocations allows to study a reactivity change from the well-established catalytic ability of polyoxoanions. The further reactivity study for these clusters is in progress.

## Author contributions

The initial manuscript draft and figures were prepared by S for his PhD research and revised by KK and YH. All the authors had final approval of the submitted version of the paper.

### Conflict of interest statement

The authors declare that the research was conducted in the absence of any commercial or financial relationships that could be construed as a potential conflict of interest.
