# Supplementary material for: Strategic Isolation of a Polyoxocation Mimicking Vanadium(V) Oxide Layered-Structure by Stacking of [H2V2O8]4− Anions Bridged by (1,4,7-Triazacyclononane)Co(III) Complexes
Source: Front Chem. 2018 Aug 28;6:375. doi: 10.3389/fchem.2018.00375 (PMC6121072; doi:10.3389/fchem.2018.00375)
Supplement: Supplementary file 1 [file Data_Sheet_1.docx]

Supplementary Material

Strategic Isolation of A Polyoxocation Mimicking Vanadium(V) Oxide Layered-Structure by Stacking of [H_2_V_2_O_8_]^4‒^ Anions Bridged by (1,4,7-Triazacyclononane)Co(III) Complexes

Sugiarto^1^, Keisuke Kawamoto^1^, Yoshihito Hayashi^1*^

^1^Department of Chemistry, Kanazawa University, Kakuma, Kanazawa, Ishikawa, Japan

*** Correspondence:**Yoshihito Hayashi
[hayashi@se.kanazawa-u.ac.jp](mailto:hayashi@se.kanazawa-u.ac.jp)

## Supplementary Figures


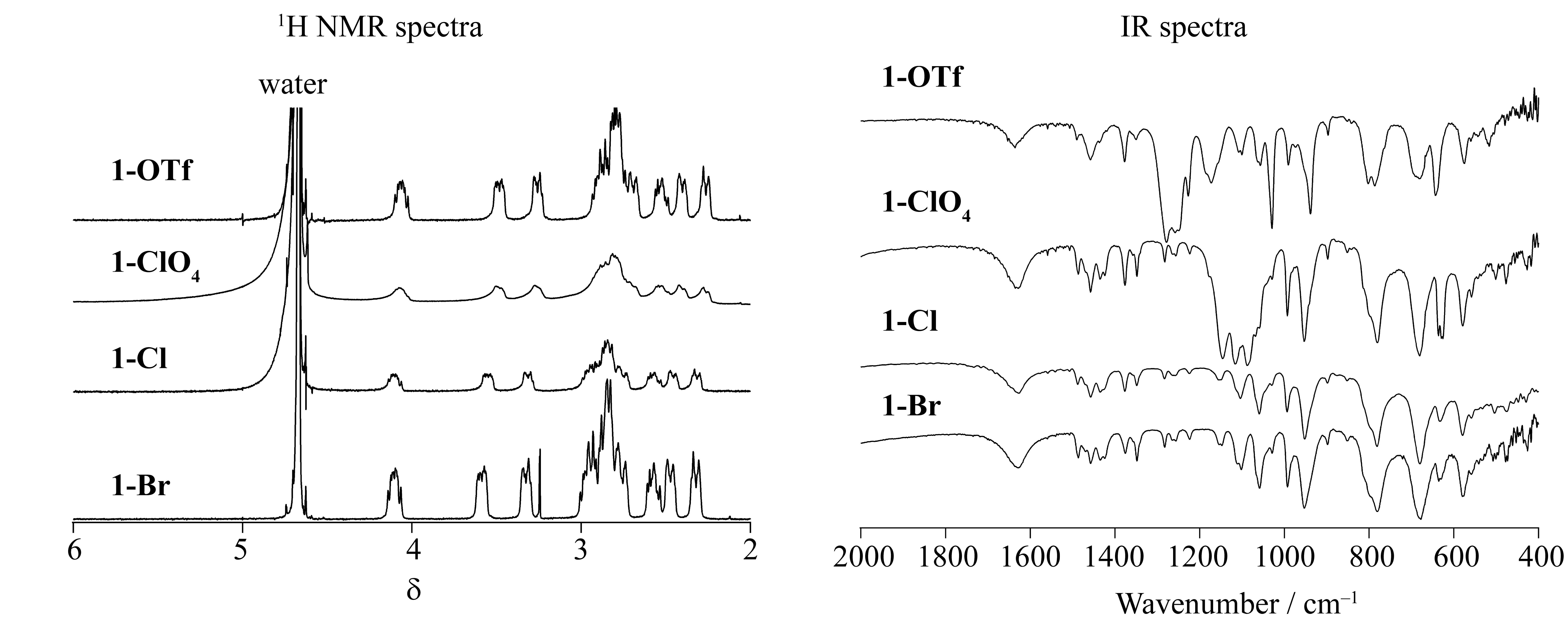


Figure S1. ^1^H NMR (in D_2_O) and IR spectra of **1-OTf**, **1-ClO_4_**, **1-Cl**, and **1-Br**.


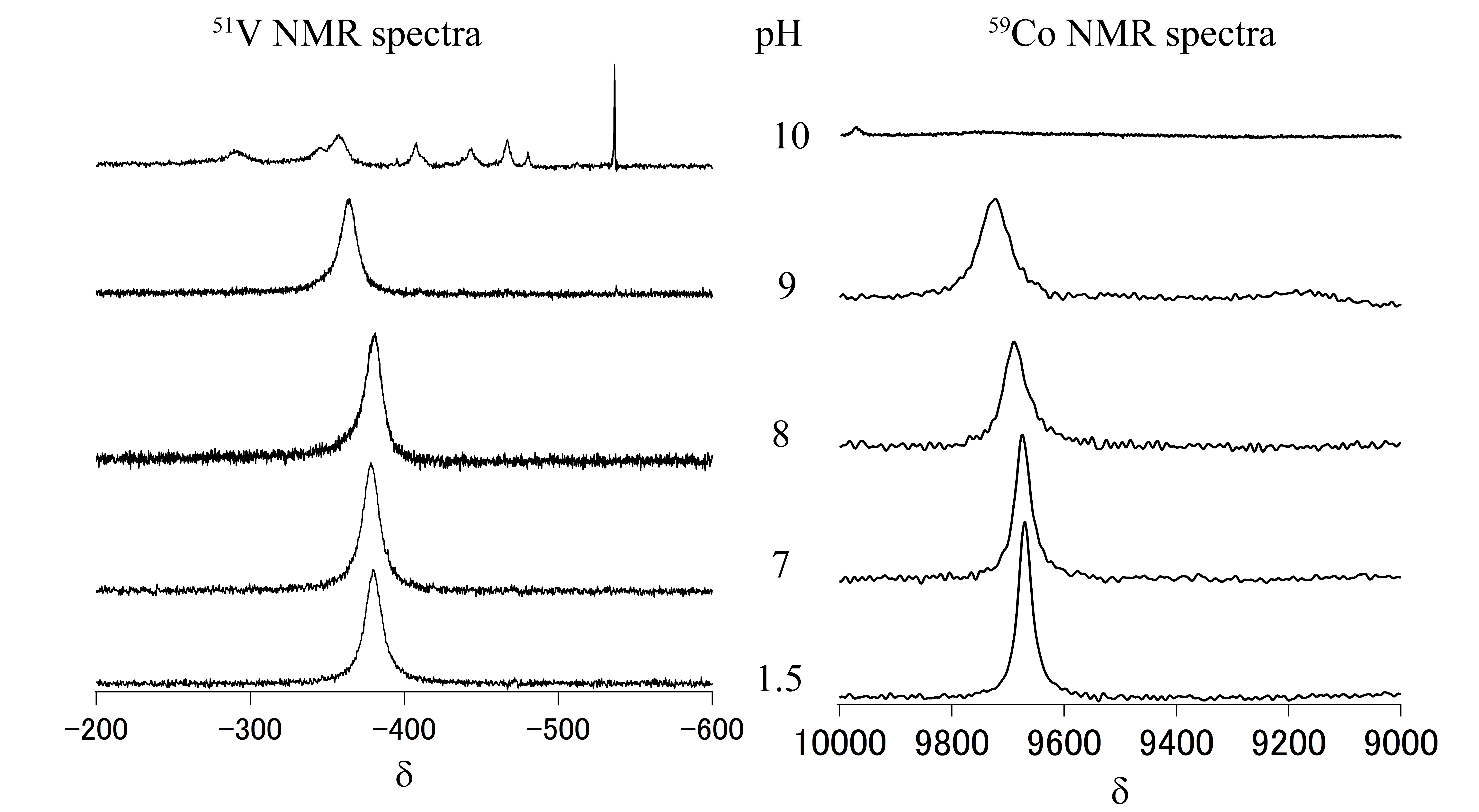


Figure S2. pH dependence of ^51^V and ^59^Co NMR spectra of cluster **1-OTf** in water.


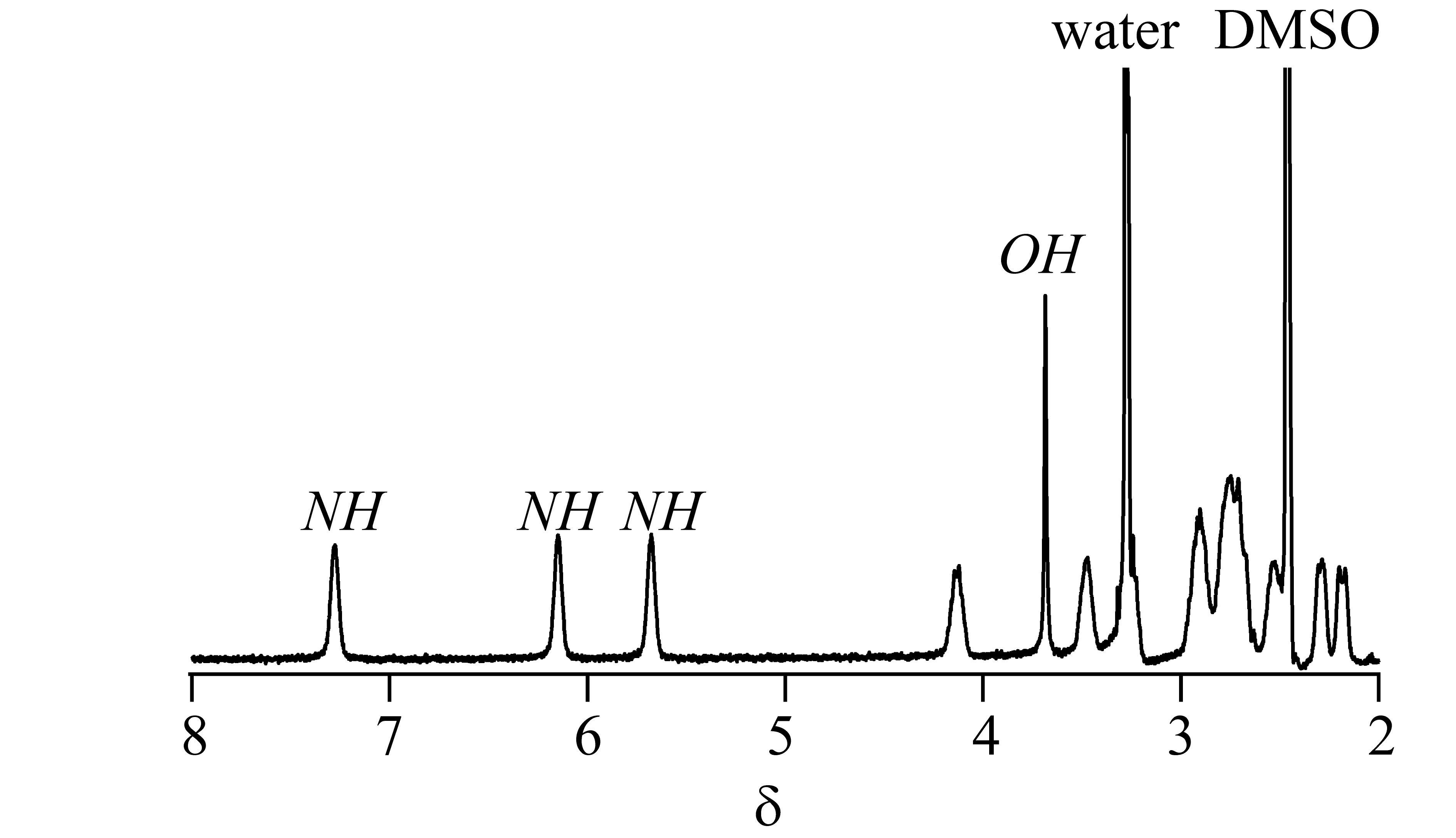


Figure S3. ^1^H NMR spectrum of **1-OTf** in DMSO-*d*_6_.


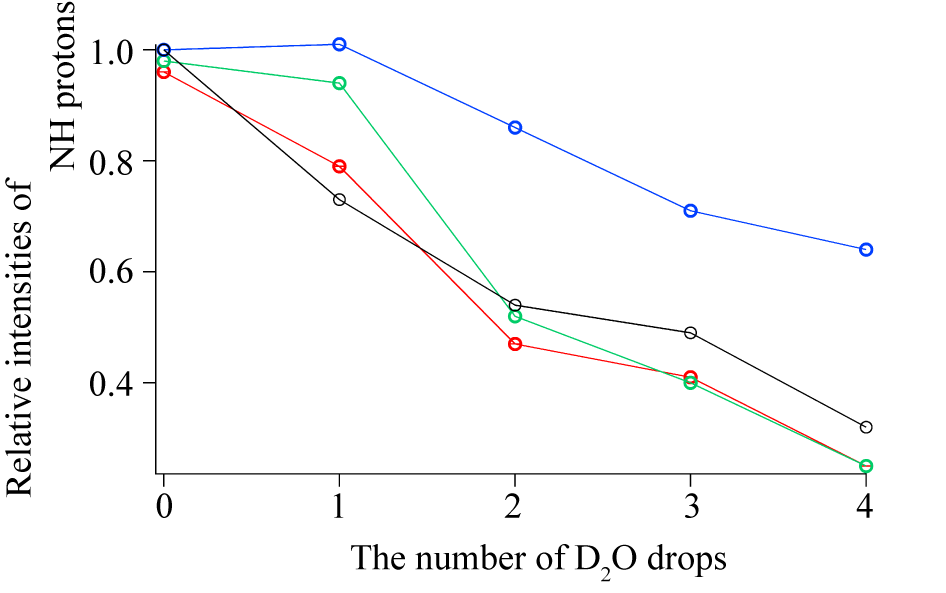


Figure S4. Decreasing of relative intensities of NH protons on cluster **1-OTf**. NH protons at 7.29, 6.15, and 5.69 ppm are shown in red, green and blue lines, respectively. The relatively sharp peak at 3.69 ppm belongs to OH hydrogen bondings and the decreasing of the intensity (black line) is similar to NH protons at 7.29 and 6.15 ppm. The NH group observed at 5.69 ppm is strongly interacted to OH bridged ligand, whereas the rest of NH and OH groups are connected to terminal oxygen ligand or crystallization water molecule as relatively week hydrogen bondings.
